# Identification of novel genetic susceptibility loci for Behçet's disease using a genome-wide association study

**DOI:** 10.1186/ar2695

**Published:** 2009-05-14

**Authors:** Yiping Fei, Ryan Webb, Beth L Cobb, Haner Direskeneli, Güher Saruhan-Direskeneli, Amr H Sawalha

**Affiliations:** 1 Department of Medicine, University of Oklahoma Health Sciences Center, 825 NE 13th Street, Oklahoma City, OK 73104, USA; 2 Arthritis & Immunology Program, Oklahoma Medical Research Foundation, 825 NE 13th Street, Oklahoma City, OK 73104, USA; 3 College of Public Health, University of Oklahoma Health Sciences Center, 825 NE 13th Street, Oklahoma City, OK 73104, USA; 4 JK Autoimmunity Inc., 755 Research Parkway, Oklahoma City, OK 73104, USA; 5 Division of Rheumatology, Department of Internal Medicine, Marmara University Medical School, Tophanelioglu Cad. 13/15, 34662, Uskudar, Istanbul, Turkey; 6 Department of Physiology, Istanbul Faculty of Medicine, Istanbul University, Capa 34093, Istanbul, Turkey; 7 US Department of Veterans Affairs Medical Center, 921 NE 13th Street, Oklahoma City, OK 73104, USA

## Abstract

**Introduction:**

Behçet's disease is a chronic systemic inflammatory disease that remains incompletely understood. Herein, we perform the first genome-wide association study in Behçet's disease.

**Methods:**

Using DNA pooling technology and the Affymetrix 500K arrays, we identified possible candidate gene associations with Behçet's disease in a cohort of 152 Behçet's disease patients and 172 healthy ethnically matched controls. Genetic loci that were identified in the pooling study were genotyped in patients and controls using TaqMan genotyping technology.

**Results:**

We identified genetic associations between Behçet's disease and single-nucleotide polymorphisms (SNPs) in *KIAA1529*, *CPVL*, *LOC100129342*, *UBASH3B*, and *UBAC2 *(odds ratio = 2.04, 2.26, 1.84, 1.71, and 1.61, respectively; *P *value = 4.2 × 10^-5^, 1.0 × 10^-4^, 3.0 × 10^-4^, 1.5 × 10^-3^, and 5.8 × 10^-3^, respectively). Among the associated SNPs, the Behçet's disease-risk allele in rs2061634 leads to substitution of serine to cysteine at amino acid position 995 (S995C) in the KIAA1529 protein.

**Conclusions:**

Using an unbiased whole-genome genetic association approach, we identified novel candidate genetic loci that are associated with increased susceptibility for Behçet's disease. These findings will help to better understand the pathogenesis of Behçet's disease and identify novel targets for therapeutic intervention.

## Introduction

Behçet's disease is a chronic relapsing systemic inflammatory disease characterized by the presence of oro-genital ulcers, cutaneous manifestations, and uveitis. The disease can also lead to vascular complications such as arterial and venous thrombosis, central nervous system vasculitis, arthritis, and gastrointestinal involvement [1]. The etiology of Behçet's disease is not fully understood. Therefore, treatment remains insufficient and relies on non-specific immunosuppressive medications, with significant side effects. Evidence for a genetic contribution to the pathogenesis of the disease comes from strong familial aggregation, the strong predominance in patients with Mediterranean or Asian ancestry, and the confirmed association with HLA-B51 in several ethnic groups [2-4].

It is estimated that the association with HLA-B51 in Behçet's disease accounts for only ~20% of the relative risk in siblings of affected individuals [1]. This suggests that other genetic elements outside the HLA region carry risk for developing Behçet's disease. Indeed, a genetic linkage study in a cohort of Behçet's disease multiplex families identified evidence for linkage (*P *≤ 0.05) on 16 chromosomal regions: 1p36, 4p15, 5q12, 5q23, 6p22-24, 6q16, 6q25-26, 7p21, 10q24, 12p12-13, 12q13, 16q12, 16q21-23, 17p13, 20q12-13, and Xq26-28 [5]. Genetic association studies performed via genotyping single-nucleotide polymorphisms (SNPs) in candidate genes have been performed. These revealed genetic associations with several genes, including *IRF1 *(interferon regulatory factor 1) [6], *TNF *(tumor necrosis factor) [7,8], and *PTPN22 *(protein tyrosine phosphatase, non-receptor type 22) [9].

Despite efforts to discover the genetic contribution to the disease etiology, with the exception of the strong association in the HLA region, other genetic associations are largely unconfirmed. We hypothesize that novel genetic loci for Behçet's disease will be identified using an unbiased genome-wide association approach. Herein, we used a DNA pooling technology to perform the first genome-wide association study in Behçet's disease. We identify five novel genetic loci that are associated with the susceptibility to develop Behçet's disease in a cohort of Turkish Behçet's disease patients and controls.

## Materials and methods

### Patients and controls

We studied a cohort of Behçet's disease patients and controls recruited at Marmara University Medical School, Istanbul, Turkey. Our cohort consisted of 152 Behçet's disease patients and 172 ethnically matched normal healthy controls. All patients fulfilled the 1990 International Study Group classification criteria for Behçet's disease [10]. DNA was extracted from peripheral blood mononuclear cells using standard techniques. All protocols were approved by the institutional review boards or the research ethics committees at Marmara University, the University of Oklahoma Health Sciences Center, and the Oklahoma Medical Research Foundation. All patients provided written informed consent.

### DNA pooling studies

For the initial discovery phase of novel genetic associations, we used a DNA pooling genotyping methodology. DNA pools were generated from cases (five pools) and controls (five pools). DNA samples were first run on a 0.8% agarose gel to examine the quality of DNA (high molecular weight) and absence of RNA contamination. The DNA quality and quantity were then determined using a NanoDrop^® ^spectrophotometer (NanoDrop Technologies, Inc., now part of Thermo Fisher Scientific Inc., Waltham, MA, USA). Three hundred twenty-two out of 324 DNA samples had a 260/280 ratio between 1.65 and 2.0 and a 260/230 ratio between 1.0 and 2.2 and were used for the pooling experiment. Samples were then serially diluted to a concentration of 17 to 23 ng/μL. Following the dilution, each DNA sample was read twice on the NanoDrop and an average was used to determine the concentration. The number of samples in each pool dictated the amount each sample contributes to the pool. The final volume of each pool was 46.5 μL. This represents a pool for each of the restriction enzymes used (15.5 μL each) and a test pool (15.5 μL). The final amount of total genomic DNA in each pool was 250 ng. After the pools were generated, the exact protocol developed by Affymetrix (Santa Clara, CA, USA) for a single DNA sample was followed. This included digestion with the appropriate restriction enzyme, ligation of linkers, amplification of targeted DNA, purification of amplified DNA, labeling, hybridization, and scanning. The Affymetrix GeneChip^® ^Human Mapping 500K Array was used. Scanning and analysis were performed using GTYPE GeneChip Genotyping Analysis Software version 4.0.0.22 and GCOS GeneChip Operating Software version 1.4.0.036. The GTYPE GeneChip Genotyping Analysis software generates the cel file that contains the signal intensity information in which allele frequencies were determined using software developed in-house by personnel at JKA Genomics (Oklahoma City, OK, USA). GeneChip Genotyping 4.0 software output was used for relative quality control assessment of detection rates (>98.5%) and allele distributions (<10% difference between pools).

### Genotyping

Candidate genes discovered in our pooling experiment were subsequently genotyped in patients with Behçet's disease and controls using a case-control genetic association approach and the TaqMan SNP Genotyping Assays (Applied Biosystems, Foster City, CA, USA). Allele frequencies and odds ratios (ORs) were determined. The Pearson chi-square values and *P *values were calculated using Haploview 4.1 [11]. Hardy-Weinberg equilibrium (HWE) *P *value measures the difference between the observed genotype frequency and the expected genotype frequency based on the observed allele frequency and was determined to examine the randomness of mating and lack of relatedness in our study population. Permutation *P *values for the allele frequency difference were calculated to correct for multiple testing using Haploview 4.1 [11].

## Results

We studied a cohort of 152 Behçet's disease patients and 172 ethnically matched controls. Our patient groups included 81 (53.3%) men and 71 (46.7%) women. The control group included 79 (45.9%) men and 93 (54.1%) women. The average age of patients at the time of enrollment was 36.4 ± 11.7 years (mean ± standard deviation, SD), and the average age for control participants was 34.2 ± 11.4 years (mean ± SD). There was no significant difference in age between patients and controls (*P *> 0.05). The clinical characteristics of our Behçet's patients included in this study are summarized in Table 1.

**Table 1:** Clinical characteristics of the Behçet's disease cohort studied

	Number (percentage)
Gender	
Males	81 (53.3%)
Females	71 (46.7%)
Clinical manifestations	
Oral ulcers	152 (100%)
Genital ulcers	115 (75.7%)
Eye disease	59 (38.8%)
Cutaneous involvement	146 (96.1%)
Gastrointestinal involvement	4 (2.6%)
Arthritis/arthralgia	79 (52%)
Neurological involvement	11 (7.2%)
Vascular involvement	44 (29%)

A total of 152 patients were included in this study.

The genome-wide scanning included pooled DNA from 152 Behçet's disease patients and 170 controls (2 DNA samples from controls did not pass DNA quality measures and were excluded from the pooling experiment). Genes with at least one SNP with an association *P *value of less than 1 × 10^-5 ^or at least 2 SNPs with an association *P *value of less than 1 × 10^-4 ^were identified as possible candidate genes for Behçet's disease and were selected for individual case-control genotyping.

We have identified 10 possible candidate genes based on the unbiased genome-wide pooling association study. Individual case-control genotyping using TaqMan SNP Genotyping Assays confirmed the genetic association between SNPs in five genes that we identify as novel genetic susceptibility loci for Behçet's disease. All SNPs had an HWE *P *value of greater than 0.05 (Table 2). Genotyping success rate was at least 96% in the SNPs genotyped, with the exception of the SNP rs4949332, which had a genotyping success rate of 90%.

**Table 2:** Genetic association between candidate single-nucleotide polymorphisms identified using DNA pooling in a cohort of Behçet's disease patients and controls

SNP (location)	Gene	Associated allele	Associated allele frequency		OR (95% CI)	*P *value	Permutation *P *value	HWE *P *value
							
			Cases n (%)	Controls n (%)				
rs11206377 (1p34)	*LOC100129342*	G	189 (66.1)	149 (51.4)	1.84 (1.32–2.58)	3.0 × 10^-4^	0.0036	0.89
rs12112050 (7p15)	*CREB5*	T	202 (67.8)	187 (63.2)	1.23 (0.87–1.72)	0.24	0.93	0.75
rs2061634 (9q22)	*KIAA1529*	G	128 (42.7)	79 (26.7)	2.04 (1.45–2.88)	4.2 × 10^-5^	0.0002	0.35
rs2875984 (8q12)	*CHD7*	T	61 (20.3)	54 (18.6)	1.12 (0.74–1.68)	0.60	1	0.87
rs317711 (7p15-p14)	*CPVL*	C	76 (25.5)	39 (13.2)	2.26 (1.47–3.45)	1.0 × 10^-4^	0.0011	0.20
rs4936742 (11q24)	*UBASH3B*	T	161 (56.7)	125 (43.4)	1.71 (1.23–2.38)	1.5 × 10^-3^	0.0195	0.84
rs4949332 (1p35)	*PUM1*	C	162 (60.9)	143 (53.0)	2.38 (0.98–1.95)	0.064	0.50	0.83
rs546550 (1p22-p21)	*ABCA4*	A	246 (84.8)	230 (78.8)	1.51 (0.98–2.31)	0.058	0.47	0.13
rs6798232 (3q23)	*PLS1*	G	139 (46.3)	125 (42.8)	1.15 (0.83–1.60)	0.39	0.99	0.68
rs9513584 (13q32)	*UBAC2*	G	128 (44.4)	95 (33.2)	1.61 (1.15–2.26)	5.8 × 10^-3^	0.064	0.94

Genotyping was performed using the TaqMan SNP Genotyping Assays in the 10 candidate single-nucleotide polymorphisms (SNPs) identified. *ABCA4*, ATP-binding cassette, sub-family A (ABC1), member 4; *CHD7*, chromodomain helicase DNA binding protein 7; CI, confidence interval; *CREB5*, cAMP responsive element binding protein 5; HWE, Hardy-Weinberg equilibrium; LL, lower limit; OR, odds ratio; *PLS1*, plastin 1; *PUM1*, pumilio homolog 1; UL, upper limit.

Among the polymorphisms studied, the SNP rs2061634 located within the gene *KIAA1529 *on chromosome 9q22 showed the strongest association with Behçet's disease in our cohort. Consequently, we propose the name 'Behçet's disease-associated gene 1' (*BDAG1*) as a synonym for *KIAA1529*. The frequency of rs2061634 risk allele (G) was 42.7% in Behçet's disease cases compared with 26.7% in controls (OR = 2.04, *P *= 4.2 × 10^-5^) (Table 2). The disease-risk allele in rs2061634 leads to a substitution of serine to cysteine at amino acid position 995 (S995C) in the KIAA1529 protein.

The SNP rs317711 located within the gene *CPVL *(carboxypeptidase, vitellogenic-like) on chromosome 7p15 had a risk allele frequency (C) of 25.5% in cases compared with 13.2% in controls (OR = 2.26, *P *= 1.0 × 10^-4^). Allele G in rs11206377 located within the gene *LOC100129342 *on chromosome 1p34 had a frequency of 66.1% in cases and 51.4% in controls (OR = 1.84, *P *= 3.0 × 10^-4^). The SNPs rs9513584 and rs4936742 located within the genes *UBAC2 *(ubiquitin-associated domain [UBA] containing 2) and *UBASH3B *(ubiquitin associated and SH3 domain containing, B) were also associated with Behçet's disease with *P *values of 5.8 × 10^-3 ^and 1.5 × 10^-3^, respectively (Table 2). However, the association between rs9513584 within *UBAC2 *and Behçet's disease is not significant after correction for multiple testing.

We next determined the genotype frequency differences between patients with Behçet's disease compared with controls. The presence of the homozygous risk genotype was significantly more frequent in patients than controls in all five of the SNPs that are associated with Behçet's disease in our study (Table 3).

**Table 3:** Genotype frequencies of the Behçet's disease-associated single-nucleotide polymorphisms identified in this study

		Number			
				
SNP	Genotype	Cases	Controls	OR (95% CI)	*P *value
rs11206377	GG	62	36	2.29 (1.39–3.78)	0.0011 (GG versus AG+AA)
	AG	66	77		
	AA	16	32		
					
rs2061634	GG	32	8	4.79 (2.13–10.77)	0.000047 (GG versus CG+CC)
	CG	66	66		
	CC	56	80		
					
rs317711	CC	12	3	4.23 (1.17–15.32)	0.018 (CC versus GC+GG)
	GC	53	34		
	GG	85	112		
					
rs4936742	TT	47	23	2.60 (1.48–4.59)	0.00076 (TT versus CT+CC)
	CT	67	79		
	CC	28	42		
					
rs9513584	GG	32	14	2.69 (1.37–5.29)	0.0033 (GG versus AG+AA)
	AG	66	70		
	AA	47	63		

Odds ratios (ORs) and *P *values were calculated for the frequency of the homozygous-risk genotype in patients compared with controls. CI, confidence interval; SNP, single-nucleotide polymorphism.

A subset analysis was performed to examine the difference in allele frequencies in clinical subsets of Behçet's disease compared with normal controls (Table 4). Interpreting these data is limited by the small number of patients studied. However, the data suggest that the risk allele in rs4936742 is more common in patients with eye involvement and with vascular involvement, suggesting that the disease-associated polymorphism in *UBASH3B *might predispose to eye and vascular complications in patients with Behçet's disease. Interestingly, the risk allele in rs2061634 (*KIAA1529*) is more common in patients without eye or vascular involvement (Table 4).

**Table 4:** Subset analysis showing genetic association between clinical subsets of patients with Behçet's disease compared with normal healthy controls

Clinical subset	SNP	Associated allele	OR	95% confidence interval		*P *value
					
				LL	UL	
Eye disease present	rs11206377	G	1.81	1.13	2.90	0.0125
	rs2061634	G	1.82	1.14	2.89	0.011
	rs317711	C	2.31	1.34	3.98	0.0023
	rs4936742	T	2.17	1.37	3.44	0.0008
	rs9513584	G	1.05	0.65	1.69	0.8403
						
Eye disease absent	rs11206377	G	1.96	1.33	2.89	0.0007
	rs2061634	G	2.26	1.54	3.33	3.01 × 10^-5^
	rs317711	C	2.26	1.41	3.63	0.0006
	rs4936742	T	1.40	0.96	2.04	0.0823
	rs9513584	G	1.97	1.34	2.88	0.0005
						
Vascular disease present	rs11206377	G	2.01	1.18	3.40	0.009
	rs2061634	G	1.47	0.88	2.46	0.1385
	rs317711	C	2.20	1.21	3.99	0.0087
	rs4936742	T	2.04	1.23	3.36	0.0049
	rs9513584	G	1.74	1.05	2.86	0.0292
						
Vascular disease absent	rs11206377	G	1.86	1.29	2.70	0.001
	rs2061634	G	2.35	1.62	3.43	6.22 × 10^-6^
	rs317711	C	2.25	1.42	3.57	0.0004
	rs4936742	T	1.54	1.07	2.21	0.0209
	rs9513584	G	1.52	1.04	2.20	0.0281
						
Both eye and vascular disease present	rs11206377	G	3.11	1.29	7.47	0.0082
	rs2061634	G	1.37	0.66	2.88	0.3987
	rs317711	C	3.29	1.52	7.12	0.0015
	rs4936742	T	2.39	1.14	5.02	0.0184
	rs9513584	G	1.24	0.60	2.59	0.5585
						
Neither eye nor vascular disease present	rs11206377	G	2.10	1.36	3.24	0.0007
	rs2061634	G	2.59	1.70	3.95	7.16 × 10^-6^
	rs317711	C	2.50	1.51	4.14	0.0003
	rs4936742	T	1.30	0.86	1.98	0.2123
	rs9513584	G	1.84	1.21	2.79	0.0042

LL, lower limit; OR, odds ratio; SNP, single-nucleotide polymorphism; UL, upper limit.

## Discussion

Behçet's disease is a chronic relapsing inflammatory disease associated with debilitating clinical consequences. The disease is more frequent in regions along the 'Silk Road'; however, cases have been reported in all ethnic groups. The pathogenesis of Behçet's disease remains incompletely understood. We have performed a genome-wide genetic association study using a cohort of Turkish Behçet's disease patients and controls. We identified novel genetic susceptibility loci for Behçet's disease in *KIAA1529*, *CPVL*, *LOC100129342*, and *UBASH3B*. A probable association with *UBAC2 *was also identified.

*KIAA1529 *and *LOC100129342 *are located on chromosome 9q22 and 1p34, respectively, and have no known function to date. The association between polymorphisms within *KIAA1529 *and *LOC100129342 *with Behçet's disease suggests a potential role for these unknown genes in regulating the immune response. *LOC100129342 *is located on chromosome 1p34 close to 1p36, a susceptibility region previously identified in families with Behçet's disease by genetic linkage studies [5]. It is possible that *LOC100129342 *explains or contributes to the genetic linkage effect in that region.

The genes *UBASH3B *and *UBAC2 *both contain a UBA, suggesting that both gene products are involved in the ubiquitination pathway. Interestingly, a polymorphism in a third ubiquitin-related gene, *SUMO4 *(small ubiquitin-like modifier 4), was associated with the risk to develop Behçet's disease in Chinese patients [12]. Ubiquitination reactions are involved in diverse biological functions, including regulating receptor tyrosine kinase signaling by modulating endocytosis and degradation of activated receptors [13]. *UBASH3B *has been shown to negatively regulate T-cell receptor signaling [14]. In mice deficient in both *UBASH3B *and *UBASH3A *(both are related proteins with 75% homology), T cells are hyper-responsive to T-cell receptor stimulation and demonstrate a marked increase in cytokine production. Furthermore, double-knockout mice had increased incidence and severity of experimental autoimmune encephalomyelitis (EAE) compared with wild-type mice [14]. These data suggest that an abnormal ubiquitination pathway might be involved in the pathogenesis of Behçet's disease.

*CPVL *gene encodes for a carboxypeptidase that belongs to a large group of proteases that cleave a single amino acid from the carboxy terminus of proteins or peptides. CPVL is expressed on human macrophages but not in other types of leukocytes [15]. The exact nature and extent of function of this carboxypeptidase are yet to be determined. A polymorphism in this gene would potentially affect the function of any of the peptides cleaved by this enzyme and therefore could interfere with normal macrophage function or alter innate immune responses.

## Conclusions

We performed a genome-wide association study and identified novel candidate genetic loci that confer increased susceptibility for Behçet's disease. Further work to replicate these findings in independent cohorts, to identify the disease-causing polymorphisms in these loci, and to determine the functional consequences of these polymorphisms will help us to better understand the pathogenesis of Behçet's disease and identify novel therapeutic targets against this disease.

## Abbreviations

CI: confidence interval; *CPVL*: carboxypeptidase, vitellogenic-like; HWE: Hardy-Weinberg equilibrium; OR: odds ratio; SD: standard deviation; SNP: single-nucleotide polymorphism; UBA: ubiquitin-associated domain; *UBAC2*: ubiquitin-associated domain containing 2; *UBASH3A*: ubiquitin associated and SH3 domain containing, A; *UBASH3B*: ubiquitin associated and SH3 domain containing, B.

## Competing interests

The authors declare that they have no competing interests.

## Authors' contributions

YF performed the acquisition of data. RW performed the acquisition and analysis of data. BLC performed the analysis of pooling data. HD and GS-D provided DNA samples, made a substantial contribution to the acquisition of data, and critically revised the manuscript. AHS designed the experiments, performed data analysis and interpretation, and wrote the manuscript. All authors read and approved the final manuscript.
